# Supplementary material for: Low resistance p-type contacts to monolayer WSe2 through chlorinated solvent doping
Source: Nat Commun. 2026 Jan 20;17:718. doi: 10.1038/s41467-025-65604-3 (PMC12820234; doi:10.1038/s41467-025-65604-3)
Supplement: Supplementary file 1 — Supplementary Information [file 41467_2025_65604_MOESM1_ESM.pdf]

Supplementary Information

Low Resistance *P*-Type Contacts to Monolayer WSe_2 through Chlorinated Solvent Doping

Lauren Hoang[1], Robert K.A. Bennett[1], Anh Tuan Hoang[2], Tara Peña[1], Zhepeng Zhang[2], Marisa Hocking[2], Ashley P. Saunders[3], Marc Jaikissoon[1], Fang Liu[3], Eric Pop[1,2,4], and Andrew J. Mannix[2,*]

[1]*Dept. of Electrical Engineering, Stanford University, Stanford, CA 94305, U.S.A.*

[2]*Dept. of Materials Science and Engineering, Stanford University, Stanford, CA 94305, U.S.A.*

[3]*Dept. of Chemistry, Stanford University, Stanford, CA 94305, U.S.A.*

[4]*Dept. of Applied Physics, Stanford University, Stanford, CA 94305, U.S.A.*

This file includes

Supplementary Figures 1-15
Supplementary Note 1
Supplementary Table 1

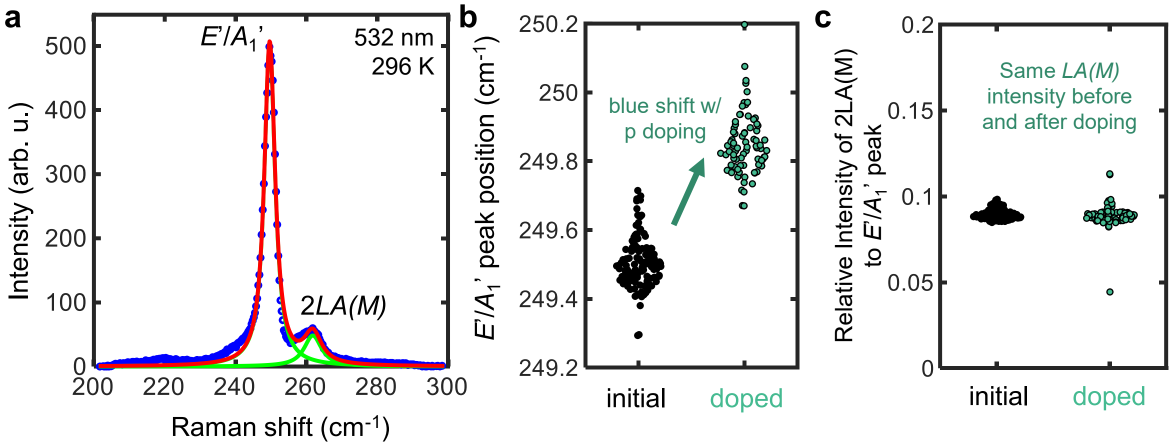

Supplementary Fig. 1 | Raman Spectroscopy on Monolayer WSe_2. a, Representative Raman spectra (blue points) and peak fitting of E'/A_1' and 2LA(M) peak using a Gaussian-Lorentzian line shape for all peaks. An iterative least-square method in MATLAB was used where the baseline of the spectra was subtracted prior to fitting. **b**, Extracted WSe_2 E'/A_1' peak position before and after chloroform doping, showing a blueshift[1–3] with doping. CVD-grown monolayer WSe_2 on SiO_2 was used for before and after doping comparison on the same WSe_2 flake. **c**, Relative intensity of the 2LA(M) peak to the E'/A_1' peak of monolayer WSe_2, showing negligible change in 2LA(M) intensity.

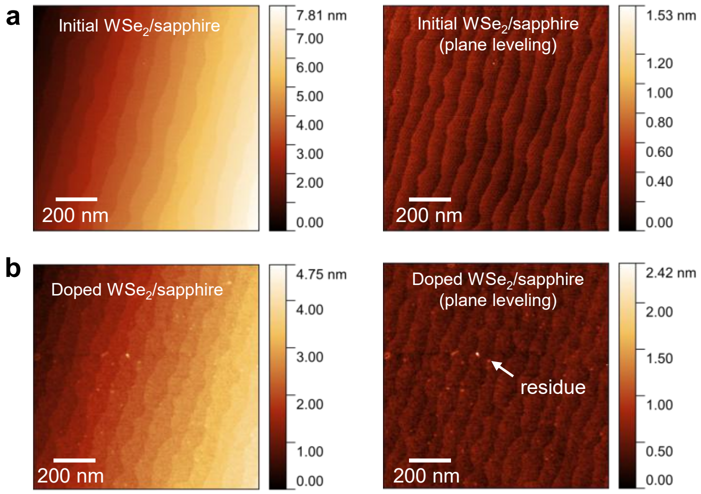

Supplementary Fig. 2 | a, Atomic Force Microscopy (AFM) image (scan size 1 × 1 μm^2) of monolayer WSe_2 grown directly on sapphire, showing low root mean square (RMS) surface roughness (RMS ~ 0.08 nm). **b**, AFM image (scan size 1 × 1 μm^2) of monolayer WSe_2 grown directly on sapphire, after doping in chloroform overnight. Some small residues can be identified on the WSe_2 flake, but there is still low surface roughness (RMS ~0.13 nm).

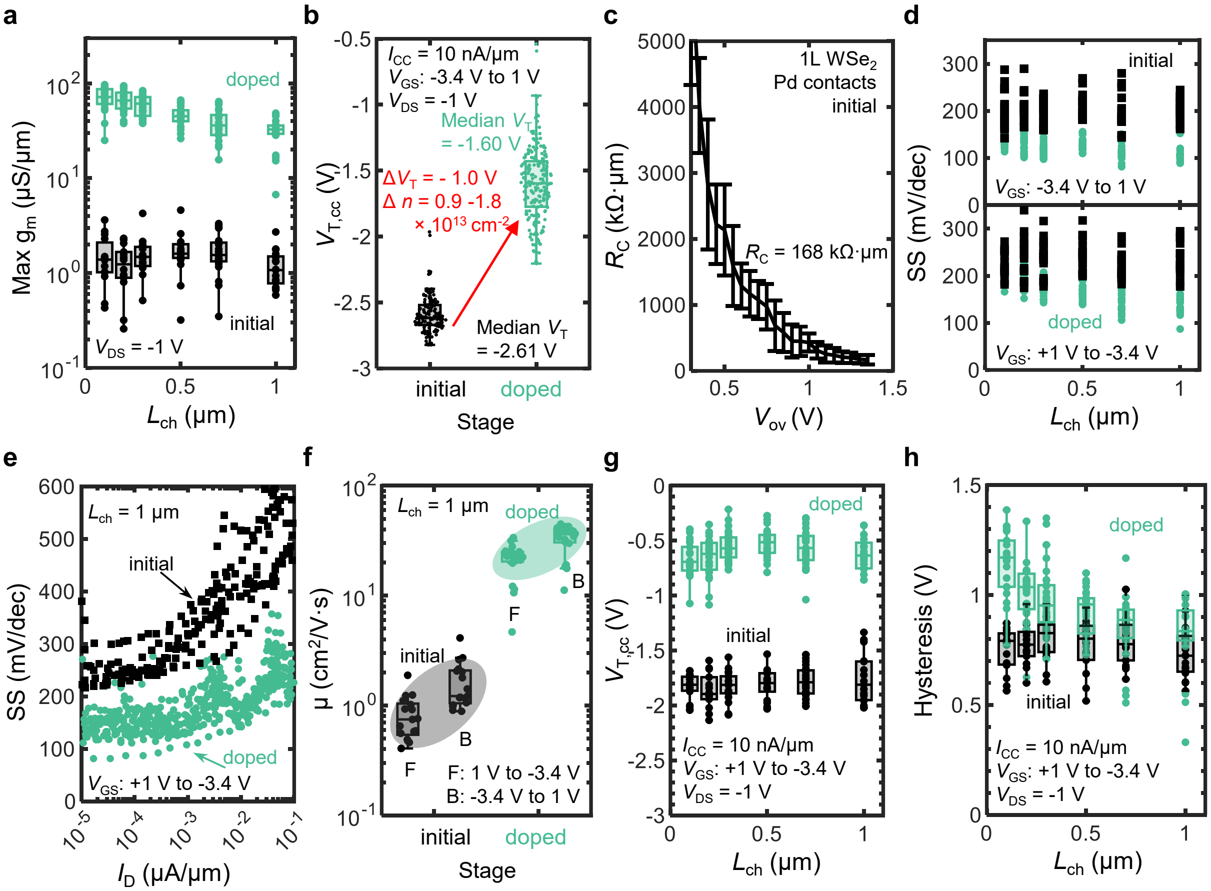

Supplementary Fig. 3 | Additional Electrical Device Analysis. a, Maximum transconductance (g_m) vs. channel length (L_{ch}) for undoped and doped devices, reaching >100 μS/μm for doped WSe_2. **b**, Constant-current threshold voltage ($V_{T,cc}$) at $I_{D,cc}$ = 10 nA/μm before and after *p*-doping. Devices show a median V_T shift of +1.0 V, corresponding to $\Delta n = C_{ox}\Delta V_T/q$ = 0.9 - 1.8 × 10^{13} cm^{-2} carriers induced (with C_{ox} = 1.38 to 2.8 μF/cm^2). **c**, Contact resistance (R_C) vs. overdrive gate voltage ($V_{ov} = |V_{GS} - V_T|$) of monolayer WSe_2 devices with Pd contacts before doping. **d**, Minimum subthreshold swing (SS) with respect to L_{ch} of doped and undoped monolayer WSe_2 devices for backward (top) and forward (bottom) V_{GS} sweep directions. **e**, SS vs. I_D in 1 μm long devices before and after doping, sweeping V_{GS} from +1 V to -3.4V (forward direction). Doped devices show lower SS for the whole sub-threshold I_D range. **f**, The mobility $\mu = g_m L_{ch}/(WC_{ox}|V_{DS}|)$ is estimated from the forward and backward V_{GS} sweeps for L_{ch} = 1 μm devices before and after doping (F and B indicate the forward and backward sweep directions, with the ranges shown in the inset). C_{ox} denotes the back-gate oxide capacitance per unit area. Mobility is extracted from long channel devices to reduce the impact of contact resistance. The initial devices are strongly contact-limited, making accurate pre-doping mobility extraction difficult. The estimated post-doping mobility is approximately 22.7 to 36.2 cm^2/(V·s) at V_{DS} = -1 V. **g**, $V_{T,cc}$ at $I_{D,cc}$ = 10 nA/μm before and after *p*-doping, sweeping from +1 V to -3.4 V. **Figure 2d** shows the counterpart $V_{T,cc}$ for the backward sweep direction from -3.4 V to 1V. **h**, Hysteresis at I_D = 10 nA/μm with respect to L_{ch}, for both undoped and doped devices. For panels a, b, f-h, a box plot was created for every group of data. The central mark of the box indicates the median, and the bottom and top edges of the box indicate the 25th and 75th percentiles, respectively.

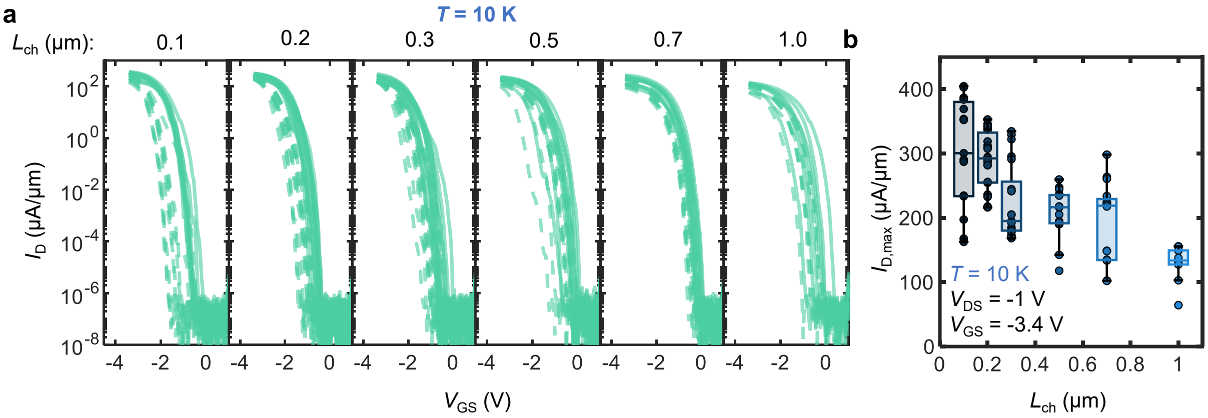

Supplementary Fig. 4 | Low Temperature Electrical Measurements of Chloroform-doped Monolayer WSe_2 Transistors. **a**, Measured I_D vs. V_{GS} at 10 K after doping for various channel length (L_{ch} = 0.1 to 1.0 μm) devices. Solid (dashed) lines indicate V_{GS} sweeps from positive (negative) to negative (positive) gate bias. **b**, Maximum drain-current $I_{D,max}$ at V_{GS} = - 3.4 V at 10 K as a function of L_{ch}. An $I_{D,max}$ of 403 μA/μm was achieved for a L_{ch} = 0.1 μm device. A box plot was created for every group of data. The central mark of the box indicates the median, and the bottom and top edges of the box indicate the 25th and 75th percentiles, respectively.

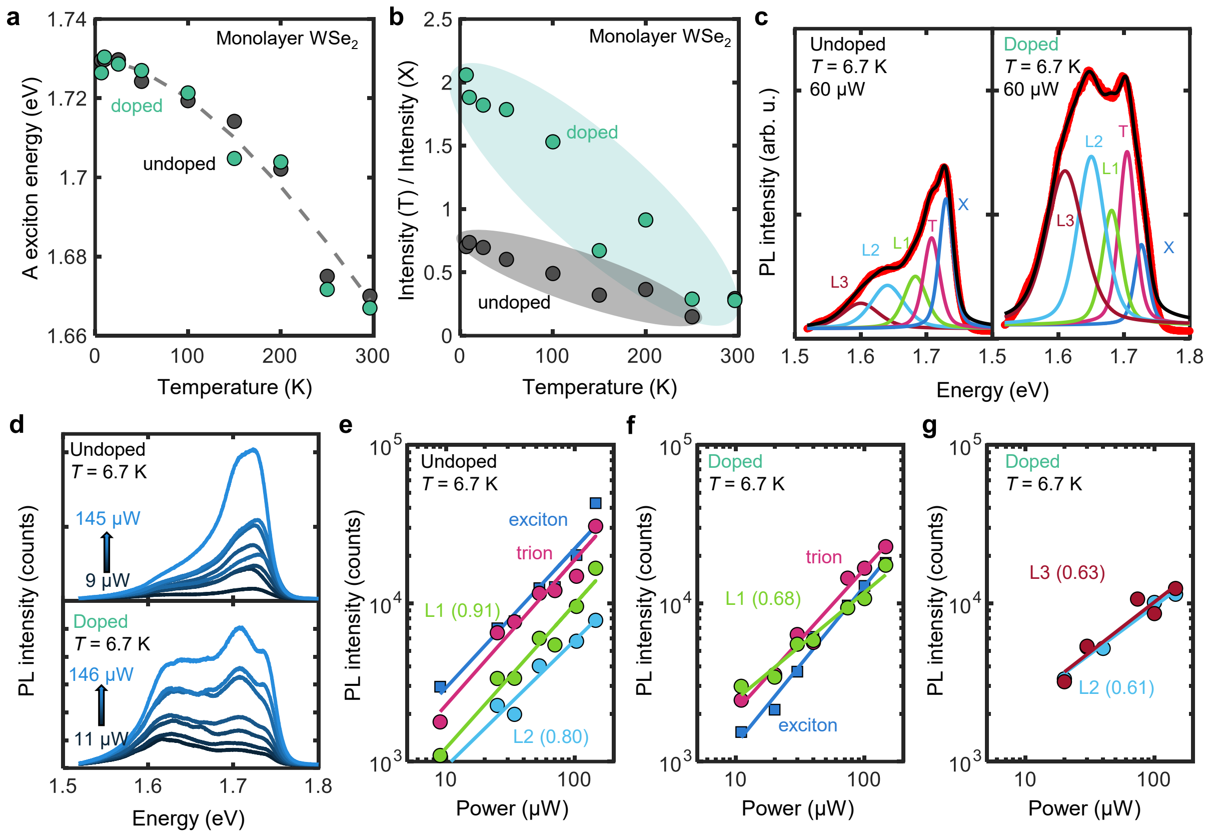

Supplementary Fig. 5 | Temperature-dependent Photoluminescence Spectroscopy. **a**, Neutral A exciton (X) energy as a function of temperature for both undoped and doped WSe_2. The exciton peak redshifts with increasing temperature, consistent with the expected band gap reduction described by the Varshni equation[4]. The data is fitted to the Varshni equation and displayed with a dashed line. **b**, Relative intensity of the trion peak to the exciton peak at different temperatures for undoped and doped WSe_2. The doped sample shows consistently higher trion intensity compared to the undoped sample. **c**, PL spectra and peak fitting of 5 peaks (X, T, L1, L2, L3) of an undoped (left) and doped (right) spectra. Noticeably, the exciton (X) intensity is higher than the trion (T), L1, L2, and L3 in the undoped sample, but lower in the doped sample. **d**, PL spectra under different laser powers collected at 6.7 K for an undoped (top) and doped (bottom) WSe_2 sample, respectively. **e**, PL intensity vs. laser power for the X, T, L1, and L2 peaks for undoped WSe_2. **f**, PL intensity vs. laser power for the X, T, and L1 peaks of doped WSe_2. The trion intensity is higher than the exciton intensity for doped WSe_2. **g**, PL intensity vs. laser power of L2 and L3 peaks of doped WSe_2. For panels e-g, the number in parenthesis indicates the power dependence (α) by fitting a power law $I \propto P^{\alpha}$ to the data, where I is the PL peak intensity for a given laser power, P. The two higher energy peaks (X, T) for both samples have near-linear fit of the peak intensity with laser power (α ~ 1), which is consistent with the exciton and trion behavior and is attributed to radiative recombination of excitons and trions. In comparison, peaks L1 - L3 exhibit sublinear power dependence, so we attribute their origin to bound excitons. We note that other peaks such as biexcitons and dark excitons have been reported to appear within this lower energy range[5,6]. The sublinear power dependence is indicative of radiative combination of electrons and holes separately localized at different spatial sites[7]. L1 - L3 peak intensities rapidly quench above 100 K as thermal stimulation perturbs the weak interaction between the defect bounded excitons[8].

a

Mulliken Population Analysis

Structure	W	Se	$CHCl_3$
Pristine WSe_2	12.970	16.515	-
Cl-facing WSe_2	12.967	16.516	26.0125
H-facing WSe_2	12.953	16.523	26.03486

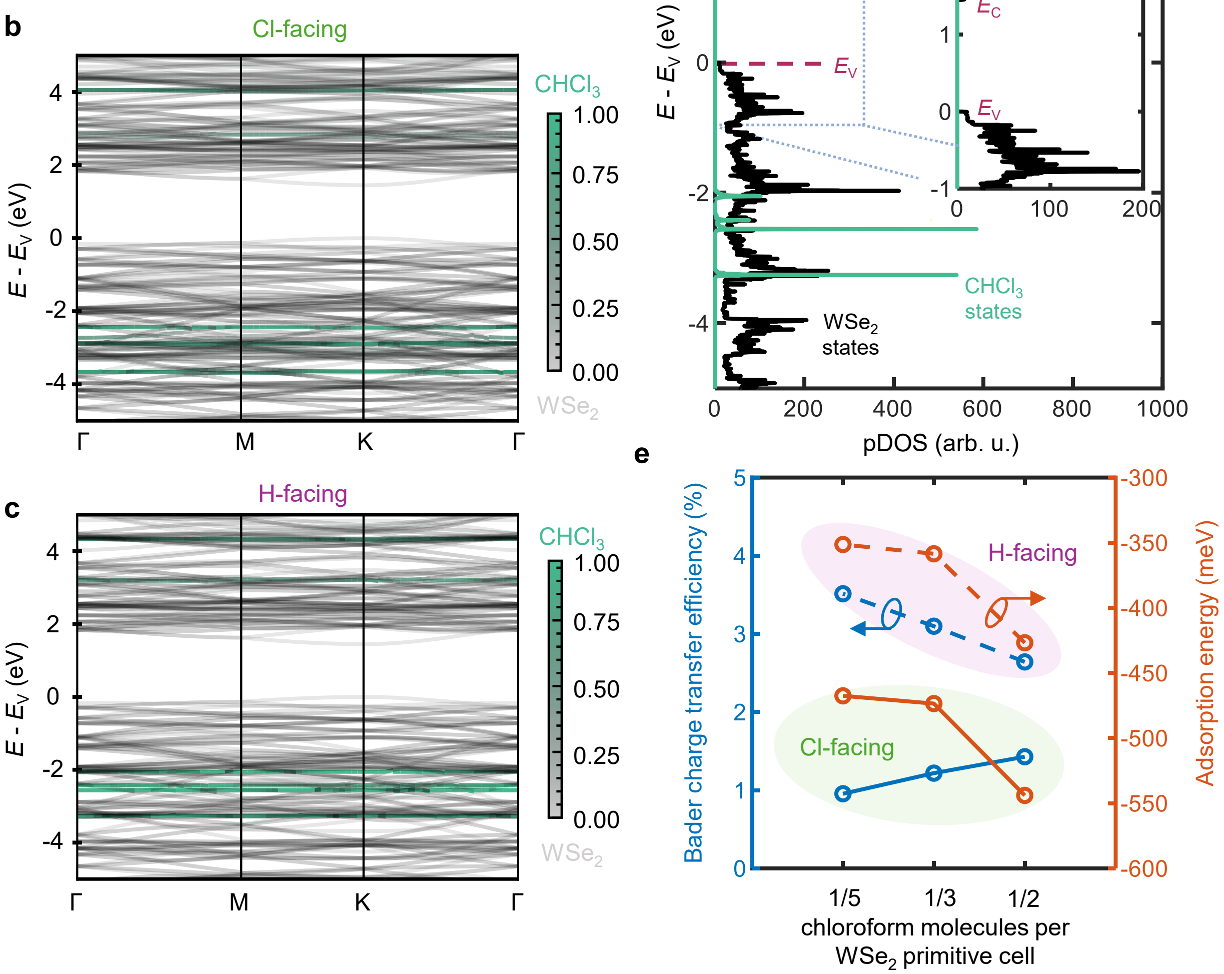

Supplementary Fig. 6 | Density Functional Theory Simulations. a, Mulliken population analysis of pristine and doped (Cl-facing and H-facing) monolayer WSe_2. The decrease in electron population of doped WSe_2 illustrates the *p*-doping of chloroform. Band structure of **b**, Cl-facing chloroform and **c**, H-facing chloroform interfaced with monolayer WSe_2. Transparent gray bands are contributions from monolayer WSe_2 and green bands are contributions from chloroform states. No chloroform states are formed in the WSe_2 band gap for both orientations. **d**, Projected density of states (pDOS) contributions from monolayer WSe_2 and chloroform to the overall DOS in the H-facing orientation. The valence band maximum E_V and conduction band minimum E_C are marked with dashed pink lines. The inset shows a zoomed-in view of the PDOS contributions around the WSe_2 bandgap. **e**, Charge transfer efficiency and adsorption energy with respect to chloroform coverage in both Cl-facing and H-facing orientations. A 2×2, 3×3, and 5×5 WSe_2 supercell were simulated with a chloroform molecule, corresponding to ½,⅓, and ⅕ chloroform molecules per WSe_2 primitive cell.

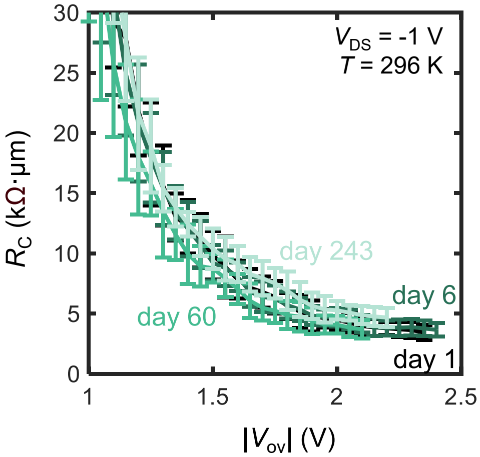

Supplementary Fig. 7 | Contact resistance (R_C) of chloroform-doped monolayer WSe_2 devices with Pd contacts 1 day, 6 days, 60 days, and 243 days (>8 months) after doping. Error bars indicate the standard error of the linear fit used for TLM extraction (fitting total resistance vs. L_{ch} at fixed V_{ov}), representing the uncertainty in the extracted R_C.

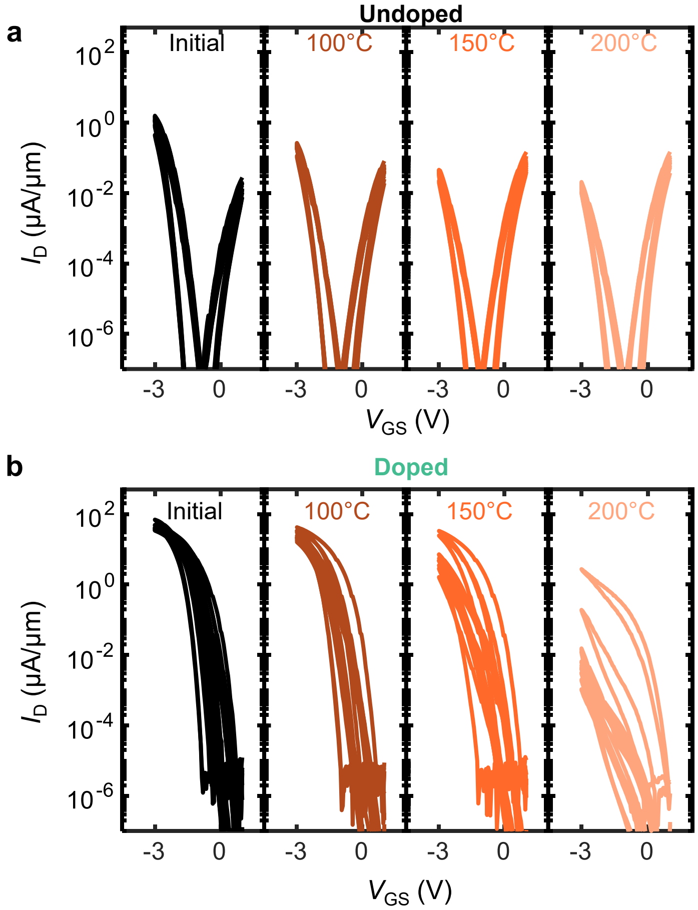

Supplementary Fig. 8 | Thermal Stability of L_{ch} = 1 μm Monolayer WSe_2 Devices. a, Measured I_D vs. V_{GS} initially and after annealing in vacuum at 100, 150, and 200°C. 9 devices were measured and plotted. **b,** Measured I_D vs. V_{GS} initially and after annealing in vacuum at 100, 150, and 200°C for chloroform-doped WSe_2. 14 devices were measured and plotted. For both plots, forward and backward sweeps are shown for all devices, and all devices demonstrate counterclockwise hysteresis.

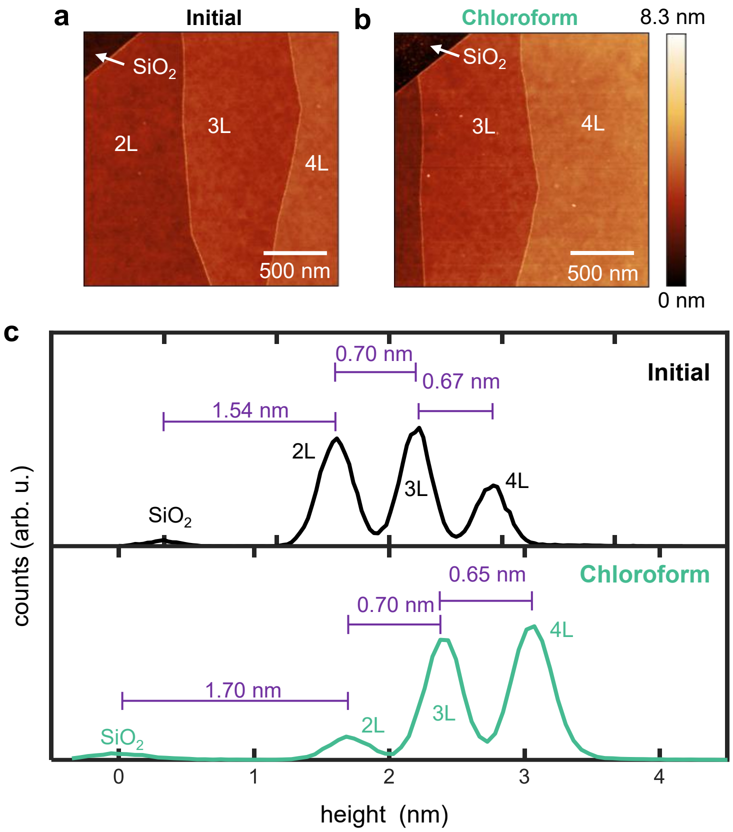

Supplementary Fig. 9 | Atomic Force Microscopy (AFM) of Exfoliated WSe_2. a, 2 × 2 μm² AFM scan of the 2-4L exfoliated WSe_2 flake in **Figure 5a** before doping. **b**, 2 × 2 μm² AFM scan of the 2-4L exfoliated WSe_2 flake in **Figure 5a** after chloroform doping. Some small particles can be identified on the flake after chloroform doping, similar to **Supplementary Fig. 2b**, but overall, there is minimal residue. **c**, Height distribution of the initial (top) and doped (bottom) AFM images in panel a and b, respectively. The peaks mark the height of the SiO_2, 2L, 3L, and 4L WSe_2 regions. There is no noticeable change in spacing between the WSe_2 layers, but the height difference between SiO_2 and 2L WSe_2 increases by > 0.15 nm. Gaussian curves were fit to the data to find the peak positions and peak separations.

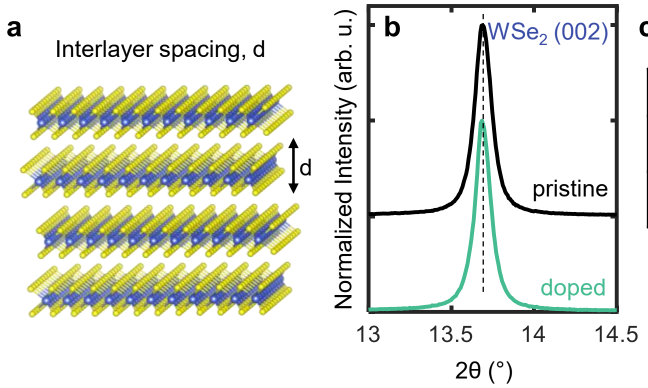

c

	2θ (°)	FWHM (°)	*d* (Å)
WSe_2 (pristine)	13.693	0.12	6.461
WSe_2 (doped)	13.689	0.11	6.463

Supplementary Fig. 10 | X-ray Diffraction (XRD) of Exfoliated Bulk WSe_2. **a**, Schematic of multilayer WSe_2 with interlayer spacing, *d*. **b**, X-ray diffraction spectra of multilayer WSe_2 (002) peak with and without chloroform doping. **c**, Calculated interlayer spacing and full-width half-maximum (FWHM) for the peaks in panel b.

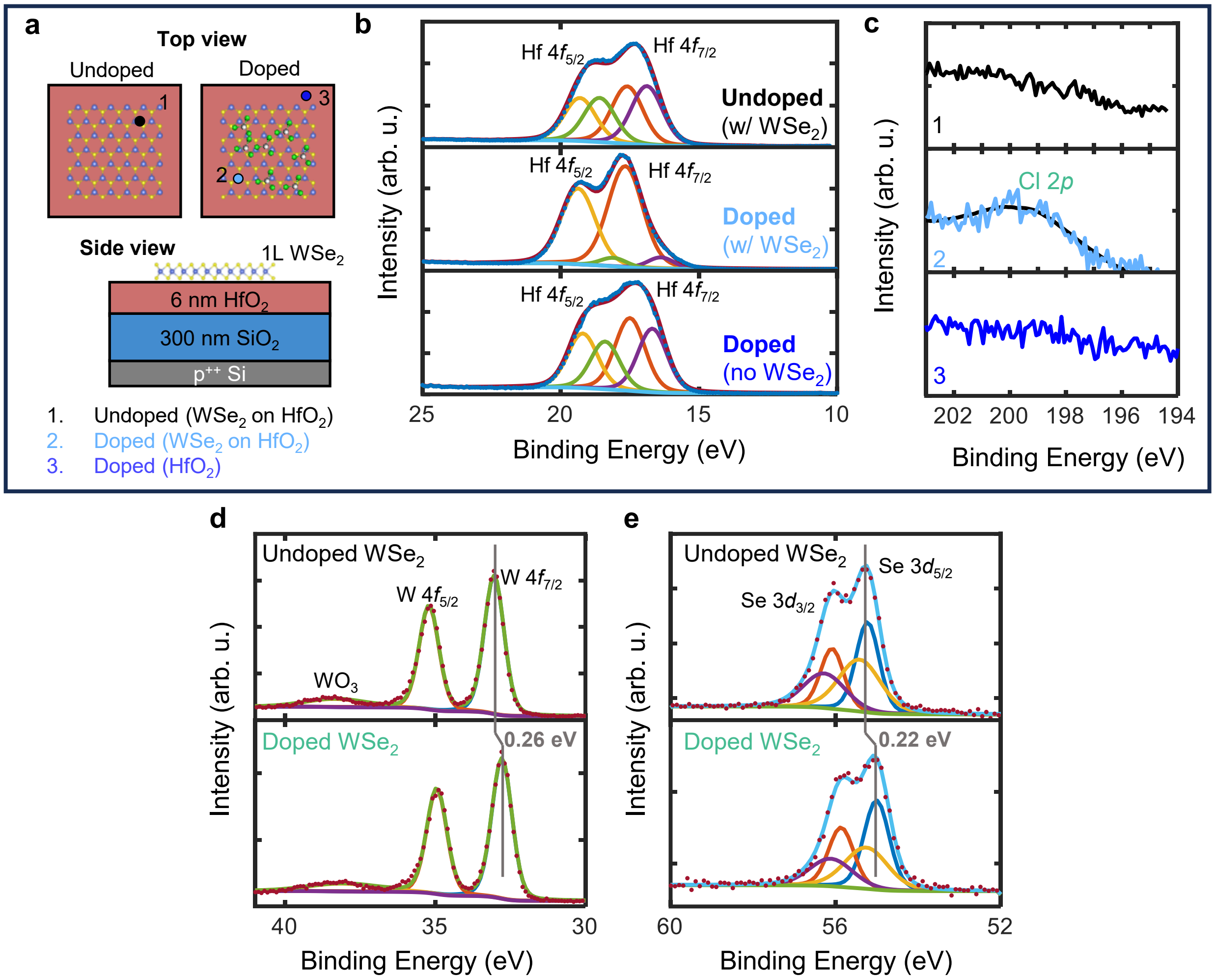

Supplementary Fig. 11 | X-ray Photoelectron Spectroscopy (XPS) of Monolayer WSe_2 on HfO_2. a, Representative schematic of 3 points probed by XPS: undoped WSe_2 on HfO_2, doped WSe_2 on HfO_2, doped HfO_2 sample with no WSe_2. HfO_2 was deposited by thermal atomic layer deposition, then underwent an O_2 plasma treatment prior to the WSe_2 transfer process (as described in Methods). **b**, XPS spectra of Hf 4*f* peaks at the 3 points located in panel a. The Hf 4*f* peaks shift to higher binding energy after exposure to chloroform. This could indicate the existence of chloroform near the HfO_2 surface, which then passivate interface traps and decrease the subthreshold swing. **c**, XPS spectra of the Cl 2*p* peak at the 3 points shown in panel a, indicating that the Cl peak exists only in the doped WSe_2 region. **d,** XPS spectra of W 4*f* peaks on undoped and doped WSe_2 on SiO_2. The W 4*f* peaks redshift by 0.26 eV, consistent with *p*-type doping. **e,** XPS spectra of Se 3*d* peaks on undoped and doped WSe_2 on SiO_2.The Se 3*d* peaks redshift by 0.22 eV. For the Se 3*d* peak fit, fitting the spectra with 2 instead of 4 peaks led to the same shift value. No new features or satellite features in the W 4*f* or Se 3*d* peaks were observed after doping. This suggests that the chloroform doping did not significantly change the chemical bonding environment for WSe_2, consistent with physisorption-dominated mechanism rather than covalent functionalization or substitution.

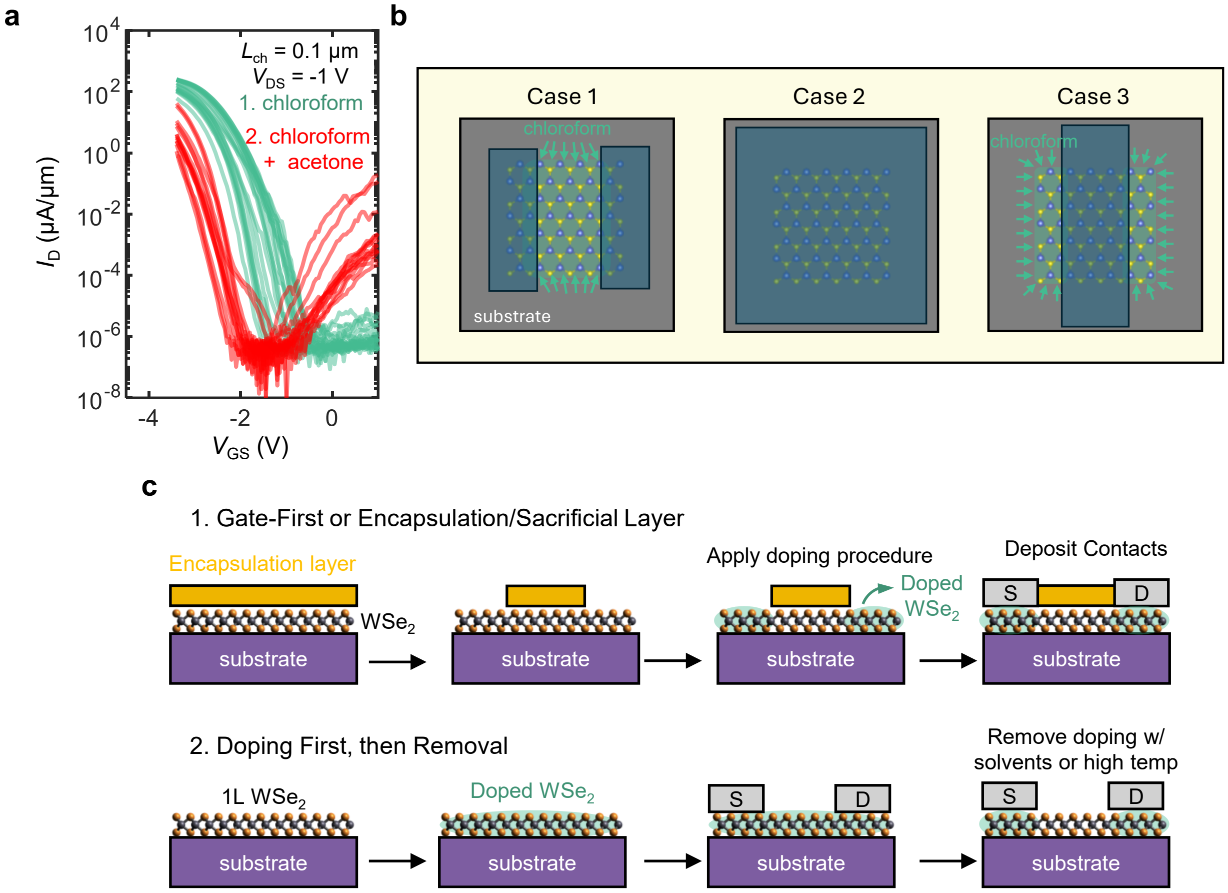

Supplementary Fig. 12 | Integration Challenges and Strategies. a, Measured I_D vs. V_{GS} curves of a monolayer WSe_2 device after doping with chloroform (green) and then after long (~8 hour) exposure to acetone (red). Devices revert back to similar currents as initial device performance (~1-10 μA/μm *p*-type drain current) and the *n*-branch re-emerges. **b,** Schematic illustrating possible chloroform intercalation pathways in WSe_2 devices with different geometries. The gray rectangle indicates the substrate, the atomic structure of a representative WSe_2 region is indicated, and blue regions indicate encapsulation by a metal or dielectric film covering the WSe_2. Chloroform intercalation pathways are indicated by green arrows. *Case 1* (left): chloroform could enter from the WSe_2 exposed edges and extend a finite distance beneath the encapsulated regions. *Case 2* (middle): In fully encapsulated geometries, no WSe_2 edges are exposed, and doping is expected to be blocked. *Case 3* (right): Partial encapsulation by oxide or metal may enable localized doping. **c**, Proposed process flows for selective-area doping with chloroform doping. Top: gate-first or encapsulation/sacrificial layer approach. Here, a sacrificial layer is first deposited, protecting the TMD from undesired doping. Doping is then selectively applied to un-protected regions. Bottom: doping then removal approach. Here, the dopant is first applied then selectively removed using high temperature processes or other solvents.

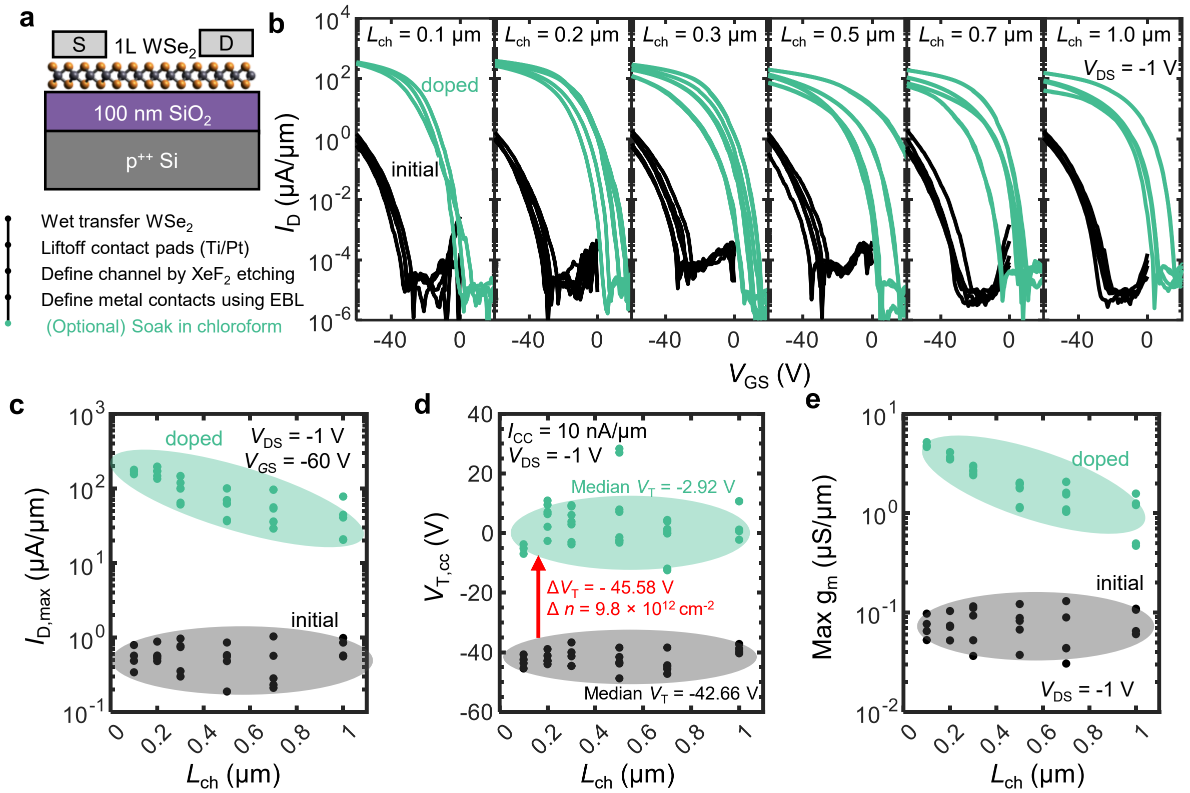

Supplementary Fig. 13 | Monolayer WSe_2 devices on 100 nm SiO_2 before and after chloroform doping. **a**, Cross-sectional schematic of WSe_2 transistor (top) and device fabrication procedure (bottom). Monolayer WSe_2 grown by chemical vapor deposition on sapphire was wet-transferred onto 100 nm SiO_2. Contact pads, channel regions, and fine contacts were defined using electron-beam lithography. Pd/Au contacts were used for fine contacts. **b**, Measured I_D vs. V_{GS} before and after doping at several channel lengths (L_{ch}) from 0.1 to 1 µm. **c**, L_{ch}-dependent statistical analysis before and after doping of maximum drain-current $I_{D,max}$ at V_{GS} = - 60 V. **d**, Threshold voltage ($V_{T,cc}$) at a constant current of 10 nA/µm before and after doping. The median V_T shift (ΔV_T) is - 45.6 V, corresponding to ~ 9.8 × 10^{12} cm^{-2} carriers induced (calculated from $\Delta n = C_{ox} \cdot \Delta V_T/q$. This closely matches the hole carriers induced on HfO_2 back-gates (**Supplementary Fig. 3b**). **e**, Maximum transconductance (g_m) vs. L_{ch} before and after doping.

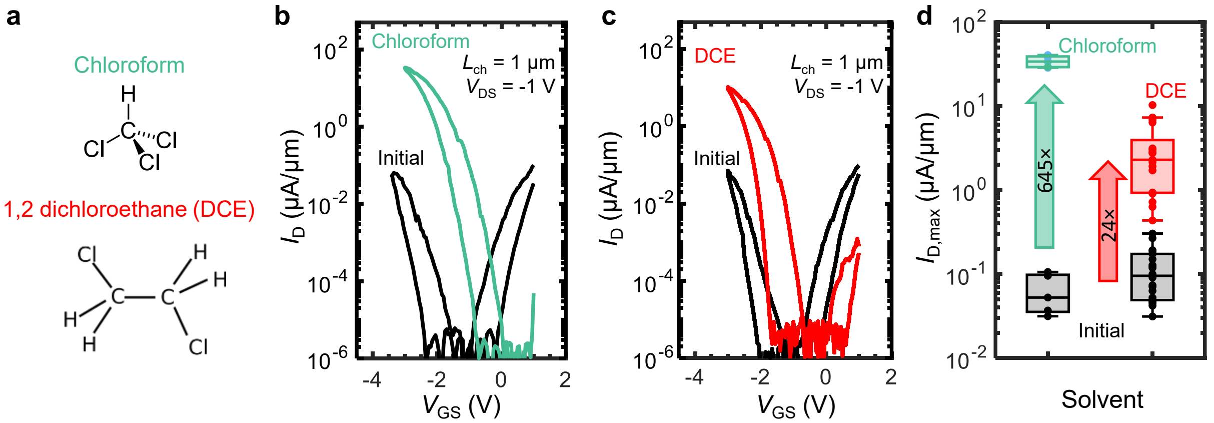

Supplementary Fig. 14 | Effect of Dichloroethane (DCE) as a *p*-type Dopant in Comparison to Chloroform. **a**, Diagram of a chloroform (top) and 1,2 dichloroethane (DCE) molecule (bottom). **b**, Measured I_D vs. V_{GS} curves of a monolayer WSe_2 device before and after doping with chloroform. Forward and backward sweeps are displayed with counter-clockwise hysteresis. **c** Measured I_D vs. V_{GS} curves of a monolayer WSe_2 device before and after doping with DCE. Forward and backward sweeps are displayed with counter-clockwise hysteresis. The devices were immersed overnight (> 8 hours) in dichloroethane (SIGMA-Aldrich, No. 34872) after device fabrication. **d**, Maximum drain-current ($I_{D,max}$) at V_{GS} = -3.0 V before and after doping with chloroform and DCE. Chloroform doped devices show >10× greater $I_{D,max}$ compared to DCE devices. All devices shown here are with L_{ch} = 1 μm. DCE increases the *hole* current of WSe_2 devices, with a median improvement in $I_{D,max}$ of ~24×, compared to the initial undoped WSe_2 devices. On the other hand, chloroform doping improves the hole current by ~ 645× on equivalent devices. The extra Cl atom in chloroform would increase the dipole moment of the dopant molecule, and thus could stabilize extra electrons, increasing the charge efficiency of chloroform. Additionally, the greater steric hinderance imposed by the larger DCE molecule suggests that it is much less likely to slip underneath the WSe_2. A box plot was created where the central mark of the box indicates the median, the bottom and the top edges of the box indicate the 25th and 75th percentiles, respectively.

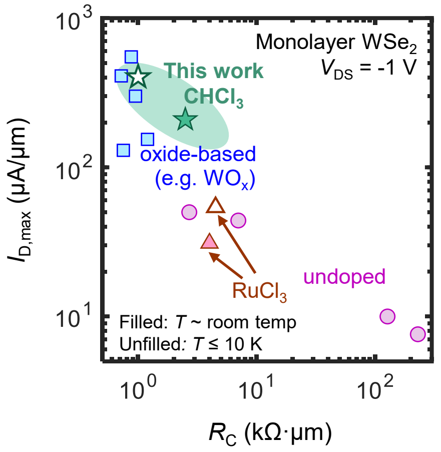

Supplementary Fig. 15 | Benchmarking maximum *p*-type current $I_{D,max}$ vs. contact resistance R_C for monolayer WSe_2 at V_{DS} = -1 V, using various contact metals and doping strategies. Circles mark results with no intentional doping[9–12], squares denote oxide-based doping (MoO_x, WO_x, NO_x)[3,13–17], and triangles label halide-based doping[18]. Filled markers indicate room temperature values and unfilled markers indicate low temperature < 10 K values. Our results with chloroform doping (stars) achieve among the highest hole currents and lowest contact resistance to date for monolayer WSe_2.

Supplementary Note 1: Estimating Initial Charge Before Chloroform Doping

To estimate the initial charge density in the WSe_2/HfO_2 stack, we use a simple analytical model[19], which derives a simple expression to estimate the threshold voltage V_T of an ultra-thin *n*-type transistor:

$$V_T = \frac{\phi_M}{q} - \frac{\chi_S}{q} - \frac{Q_{ss}}{C_{ox}} + \frac{Q_{sd}}{C_{ox}} \qquad (1)$$

where ϕ_M is the metal work function, χ_S is the semiconductor electron affinity, q is the elementary charge, and Q_{ss} is the interfacial charge density. Q_{sd} is the depletion charge density, whose magnitude is proportional to the doping concentration (and is positive if the semiconductor is *p*-doped and negative if *n*-doped). We define the total initial (i.e., before doping with chloroform) charge density $Q_{total} = Q_{sd} - Q_{ss}$, and rearrange the above equation to obtain:

$$Q_{total} = Q_{sd} - Q_{ss} = \left(V_T + \frac{\chi_S}{q} - \frac{\phi_M}{q}\right) C_{ox} \qquad (2)$$

To estimate Q_{total}, we use the measured transfer characteristics of the initial devices as seen in **Figure 2b.** The as-fabricated devices exhibit ambipolar *n*-type behavior, with the *n*-branch emerging near V_{GS} = 0 V. We take an *n*-type $V_T \approx 1$ V at L_{ch} = 1 μm from **Figure 2b** and $\chi_S = 3.9$ eV[20] for monolayer WSe_2. The work function of thin (8 nm) Pt films is $\phi_M \approx 5.5$ eV[21], though 2 nm Ti in the gate stack could lower this; thus, we estimate that ϕ_M could be between 5.0 to 5.5 eV. With these values, and assuming an EOT of 2.4 nm (including the contribution from the van der Waals gap), we estimate $Q_{total} \approx -1.4 \times 10^{-7}$ to -8.6×10^{-7} C·cm^{-2}, or approximately 9×10^{11} to 5×10^{12} charges per cm^2. Thus, upon chloroform doping, the induced hole density of ~10^{13} cm^{-2} (**Supplementary Fig. 3b**) compensates this initial electron density, dominating the final carrier concentration and shifting the device to *p*-type operation.

We stress that this is a very approximate range of Q_{total}, due to both approximations made when deriving equation (1) and uncertainties in the quantities used when calculating Q_{total} in equation (2). Further, we emphasize that the range calculated above is not merely the initial background doping concentration; rather, it is the difference between the charge density from doping and the immobile charge at or near the semiconductor/oxide interface. Although we are unable to separate these two terms, this range of Q_{total} still indicates an effective initial charge density that we anticipate will help others reproduce the results of this work.

While the precise initial carrier concentration may vary between WSe_2 sources, its influence on the final carrier density after doping is expected to be limited, as the doping process is primarily governed by adsorption and charge transfer rather than by the preexisting doping level. Consistent with this, the >100× increase in current after doping suggests that the induced carrier population substantially exceeds the initial carrier density.

Supplementary Table 1: Benchmarking the Electrical Performance of Monolayer WSe_2

Ref	Contact metal	Dopant	L_{ch} (nm)	I_{on} (μA/μm), $\|V_{DS}\|$ = 1 V	I_{on}/I_{off}	R_C (kΩ·μm)*	Stability (time, temperature)
9	Pd	-	600	10	10^8	127	-
10	Ru	-	200	50	2×10^7	2.7	-
22	Ru	-	100	100	10^8	-	-
11	Pt	-	1500	7.6	2×10^5	229	-
23	Pt/Au 10/80 nm	-	700	108.1	2×10^8	-	-
24	Ru/Au	-	70	247	10^8	-	-
25	Pt/Au 20/60 nm	-	200	15	10^7	-	-
26	TiO_2/Ru	-	140	100	10^8	-	-
27	Ru	-	50	92	10^8	-	-
13	Ti/Pd/Ni 1/30/30 nm	NO (175°C, 4 h)	65	300	2×10^6	0.95	16 days
			85	213			
			180	153			
			380	124			
14	Sb/Pt 10/12 nm	10 nm MoO_x	100	130	-	0.75	-
		-	100	30	10^6	-	-
28	Sb/Pt 10/12 nm	MoO_x	100	170	10^7	-	-
		-	100	66	10^7	-	-
29	Pd	WO_x	500	82	10^7	~1	-
		-	1000	27.6	10^7		-
15	Pd	5 nm MoO_x	50	410	$\sim10^6$	0.72	-
		-	200	170	$\sim10^7$		-
16	Ti/Pt/Au 0.5/30/30nm	2× WO_x, NO	55	550	$\sim10^9$	0.875	-
			180	188			-
			380	105			-
			780	82			-
		-	55	20	$\sim10^8$	-	-
17	Ti/Pt/Au 0.5/30/30nm	NO	55	300	10^9	0.875	24 days
30	Pd 25 nm	$HAuCl_4$ (50mM)	5500	100	10	-	-
		$HAuCl_4$ (5mM)	5500	8	10^7	-	-
3	Pd/Au 5/50 nm	WO_xSe_y	50	154	4×10^7	1.2	-
18	few layer graphene	α-$RuCl_3$	500	31	10^9	4.0<br>4.5 (at 10 K)	-
31	few layer graphene	α-$RuCl_3$	-	-	-	1.7 (at 300 mK, uniform doping), 20 (at 300 mK contact doping)	-
	Pt	α-$RuCl_3$	-	-	-	~ 36 (at 1.5 K)	-
**This Work**	Pd/Au 20/20 nm	$CHCl_3$	1000	119.2	10^{10}	2.5<br>1.0 (at 10 K)	243 days (8 months) 150 °C vacuum anneal
			700	153	10^{10}		
			500	163.2	10^{10}		
			300	184.9	10^{10}		
			200	201.5	10^{10}		
			100	208.7	10^{10}		

*R_C value given at room temperature if not otherwise stated

Blue text indicates *p*-type doping was implemented in the work

Supplementary References

1. Kang, D. H. *et al.* Controllable nondegenerate p-type doping of tungsten diselenide by octadecyltrichlorosilane. *ACS Nano* **9**, 1099–1107 (2015).
2. Borah, A., Nipane, A., Choi, M. S., Hone, J. & Teherani, J. T. Low-Resistance p-Type Ohmic Contacts to Ultrathin WSe_2 by Using a Monolayer Dopant. *ACS Appl. Electron. Mater.* **3**, 2941–2947 (2021).
3. Chen, S., Zhang, Y., King, W. P., Bashir, R. & van der Zande, A. M. Extension Doping with Low-Resistance Contacts for P-Type Monolayer WSe_2 Field-Effect Transistors. *Adv. Electron. Mater.* **11**, 2400843 (2025).
4. Varshni, Y. P. Temperature dependence of the energy gap in semiconductors. *Physica* **34**, 149–154 (1967).
5. Li, Z. *et al.* Revealing the biexciton and trion-exciton complexes in BN encapsulated WSe_2. *Nat. Commun.* **9**, 3719 (2018).
6. Tang, Y., Mak, K. F. & Shan, J. Long valley lifetime of dark excitons in single-layer WSe_2. *Nat. Commun.* **10**, 4047 (2019).
7. Huang, J., Hoang, T. B. & Mikkelsen, M. H. Probing the origin of excitonic states in monolayer WSe_2. *Sci. Rep.* **6**, 22414 (2016).
8. Wu, Z. *et al.* Defects as a factor limiting carrier mobility in WSe_2: A spectroscopic investigation. *Nano Res.* **9**, 3622–3631 (2016).
9. Dorow, C. *et al.* Advancing Monolayer 2-D nMOS and pMOS Transistor Integration from Growth to Van der Waals Interface Engineering for Ultimate CMOS Scaling. *IEEE Trans. Electron Devices* **68**, 6592–6598 (2021).
10. O'Brien, K. P. *et al.* Advancing 2D Monolayer CMOS Through Contact, Channel and Interface Engineering. in *2021 IEEE International Electron Devices Meeting (IEDM)* 7.1.1-7.1.4 (IEEE, 2021). doi:10.1109/IEDM19574.2021.9720651.
11. Wang, Y. *et al.* P-type electrical contacts for 2D transition-metal dichalcogenides. *Nature* **610**, 61–66 (2022).
12. Chou, S.-A. *et al.* Large-scale alkali-assisted growth of monolayer and bilayer WSe_2 with a low defect density. *Nat. Commun.* **16**, 2777 (2025).
13. Chiang, C.-C. *et al.* Air-Stable P-Doping in Record High-Performance Monolayer WSe_2 Devices. *IEEE Electron Device Lett.* **43**, 319 (2022).
14. Chou, A.-S. *et al.* High-Performance Monolayer WSe_2 p/n FETs via Antimony-Platinum Modulated Contact Technology towards 2D CMOS Electronics. in *2022 International Electron Devices Meeting (IEDM)* 7.2.1-7.2.4 (IEEE, 2022). doi:10.1109/IEDM45625.2022.10019491.
15. Chou, A.-S. *et al.* Status and Performance of Integration Modules Toward Scaled CMOS with Transition Metal Dichalcogenide Channel. in *2023 International Electron Devices Meeting (IEDM)* 1–4 (IEEE, 2023). doi:10.1109/IEDM45741.2023.10413779.
16. Lan, H.-Y., Tripathi, R., Liu, X., Appenzeller, J. & Chen, Z. Wafer-scale CVD Monolayer WSe_2 p-FETs with Record-high 727 μA/μm I_{on} and 490 μS/μm g_{max} via Hybrid Charge Transfer and Molecular Doping. in *2023 International Electron Devices Meeting (IEDM)* 1–4 (IEEE, 2023). doi:10.1109/IEDM45741.2023.10413736.
17. Lan, H.-Y. *et al.* Uncovering the doping mechanism of nitric oxide in high-performance P-type WSe_2 transistors. *Nat. Commun.* **16**, 4160 (2025).
18. Xie, J. *et al.* Low Resistance Contact to P-Type Monolayer WSe_2. *Nano Lett.* **24**, 5937–5943 (2024).

19. Jackson, T. Thinking MOSFETs. *IEEE Trans. Electron Devices* **72**, 1520–1522 (2025).
20. Liu, W., Cao, W., Kang, J. & Banerjee, K. (Invited) High-Performance Field-Effect-Transistors on Monolayer WSe_2. *ECS Trans.* **58**, 281–285 (2013).
21. Lee, W.-J. *et al.* Atomic Layer Deposition of Pt Thin Films Using Dimethyl (*N*, *N*-Dimethyl-3-Butene-1-Amine- *N*) Platinum and O_2 Reactant. *Chem. Mater.* **31**, 5056–5064 (2019).
22. Maxey, K. *et al.* 300 mm MOCVD 2D CMOS Materials for More (Than) Moore Scaling. in *2022 IEEE Symposium on VLSI Technology and Circuits (VLSI Technology and Circuits)* 419–420 (IEEE, 2022). doi:10.1109/VLSITechnologyandCir46769.2022.9830457.
23. Kim, K. S. *et al.* Non-epitaxial single-crystal 2D material growth by geometric confinement. *Nature* **614**, 88–94 (2023).
24. Penumatcha, A. *et al.* High Mobility TMD NMOS and PMOS Transistors and GAA Architecture for Ultimate CMOS Scaling. in *2023 International Electron Devices Meeting (IEDM)* 1–4 (IEEE, 2023). doi:10.1109/IEDM45741.2023.10413662.
25. Wang, X., Xiong, X., Shi, X., Gu, C. & Wu, Y. Optimized electrical properties of p-type field-effect transistors based on WSe_2 grown at moderate temperatures. *Appl. Phys. Lett.* **123**, (2023).
26. Dorow, C. J. *et al.* Exploring manufacturability of novel 2D channel materials: 300 mm wafer-scale 2D NMOS & PMOS using MoS_2, WS_2, & WSe_2. in *2023 International Electron Devices Meeting (IEDM)* 1–4 (IEEE, 2023). doi:10.1109/IEDM45741.2023.10413874.
27. Mortelmans, W. *et al.* Record Performance in GAA 2D NMOS and PMOS Using Monolayer MoS_2 and WSe_2 with Scaled Contact and Gate Length. in *2024 IEEE Symposium on VLSI Technology and Circuits (VLSI Technology and Circuits)* 1–2 (IEEE, 2024). doi:10.1109/VLSITechnologyandCir46783.2024.10631395.
28. Lin, Y.-T. *et al.* Antimony–Platinum Modulated Contact Enabling Majority Carrier Polarity Selection on a Monolayer Tungsten Diselenide Channel. *Nano Lett.* **24**, 8880–8886 (2024).
29. Hung, T. Y. T. *et al.* pMOSFET with CVD-grown 2D semiconductor channel enabled by ultra-thin and fab-compatible spacer doping. in *2022 International Electron Devices Meeting (IEDM)* vols 2022-December 7.3.1-7.3.4 (IEEE, 2022).
30. Ho, P. H. *et al.* High-Performance WSe_2 Top-Gate Devices with Strong Spacer Doping. *Nano Lett.* **23**, 10236–10242 (2023).
31. Pack, J. *et al.* Charge-transfer contacts for the measurement of correlated states in high-mobility WSe_2. *Nat. Nanotechnol.* **19**, 948–954 (2024).
